# Editorial: Insights in bioprocess engineering 2021/22: novel developments, current challenges, and future perspectives

**DOI:** 10.3389/fbioe.2023.1237925

**Published:** 2023-06-29

**Authors:** Manfred Zinn, Ligia R. Rodriques

**Affiliations:** ^1^ School of Engineering, Institute of Life Technology, University of Applied Sciences Western Switzerland, Sion, Switzerland; ^2^ CEB–Centre of Biological Engineering, University of Minho, Braga, Portugal; ^3^ LABBELS–Associate Laboratory, Braga/Guimarães, Portugal

**Keywords:** bioprocess engineering, marine microbiome, bioreactors, PAT, biological loop control, chromatography

We are now entering the third decade of the 21st century, and especially in the last years, the achievements made by scientists in the field of biotechnology have been exceptional, leading to major advancements. In this Research Topic, five articles are introduced that represent current developments in bioprocess engineering, such as the exploration of the marine microbiome, tailored bioreactor technologies, the modern process control using process analytical technology or novel biological control loops in cell engineering, and simulation models in downstream processing using chromatography.

The article by Rodrigues and de Carvalho, “Cultivating marine bacteria under laboratory conditions: Overcoming the “unculturable” dogma”, addresses an important problem in marine biotechnology. Many microbial isolates from the marine environment have been identified by scientists to have significant potential as a biocatalyst for pharmaceuticals and agro- and fine chemicals. Of advantage over traditional production strains are their high salt tolerance, meaning saline water instead of sweet water can be used, thus significantly reducing the downstream processing and waste handling. Interestingly, the authors challenge the well-known “great plate anomaly” postulated by Staley and Konopka (1985), which states that less than 1% of the total direct count can be heterotrophically cultured using plating techniques. In a detailed study, Rodrigues and de Carvalho verified this statement with a profound field analysis of a marine rock pond with high salt content and exposed to high UV irradiation. They compared the culture-independent techniques (fluorescence microscopy combined with live/dead or Syto 9 staining and metagenomics) with the culture-dependent techniques (12 marine media with different nutrient concentrations). The authors found four taxonomic orders that were not detected by the metaxonomic approach and that only could be detected by cultivation on agar plates. Overall, they were able to heterotrophically cultivate 45% of the marine strains.

The coronavirus disease in 2019 clearly showed how essential a fast production of vaccines is. However, the biosynthesis of such a valuable product without suitable and high-performing bioreactors is leading to a critical situation. A suitable approach is proposed by Fang et al. “Application of bioreactor technology for cell culture-based viral vaccine production: Present status and future prospects”. This scientific review summarizes what animal cell lines have been engineered for what vaccine products, and it also critically evaluates different types of cell culture bioreactors, namely, mechanical stirring bioreactors, fixed and fluidized bed bioreactors, airlift bioreactors, hollow fiber bioreactors, and disposable bioreactors, with respect to their advantages and disadvantages. Of particular interest are the descriptions of the large-scale cultures of different types of viruses (SARS-CoV-2, influenza virus, tropical virus, enterovirus, and rabies virus). Finally, it is concluded that the design of production processes is slow because there is a lack of quantitative data and suitable, functional computer software to simulate the bioreactor’s performance and integration into a production process.

The beneficial implementation of process analytical technology (PAT) is demonstrated by Graf et al. in their original article “A novel approach for non-invasive continuous in-line control of perfusion cell cultivations by Raman spectroscopy”. They could show that the glucose concentration could be monitored and successfully used to control its concentration to 4 and 1.5 gL^-1^ with a Raman probe located in the cell-free perfusion permeate. The regulation was found to be very accurate and independent of the bioreactor scale (two or 20 L), thus facilitating scale-up problems and opening a wide range of potential applications. This PAT approach yielded high productivity and a high viability above 90%.

The review article by Stefanov and Fussenegger, “Biomarker-driven feed-back control of synthetic biology systems for next-generation personalized medicine”, gives an overview of cell signaling concepts for closed-loop compatible cell therapies to treat complex diseases. This remarkable article explains the new basic concepts and strategies for difficult-to-treat diseases and how they can be controlled to improve the quality of life and therapeutical compliance of patients. The described approaches share the interactions of biomarkers and their utilization as controllers for the therapeutic response, which follows the general and traditional principle of “sensor, processor, and effector”. Interestingly, all the cited strategies have already been tested in animal models.

Finally, Bernau et al. reviewed an important aspect of downstream processing in “The use of predictive models to develop chromatography-based purification processes”. There are many parameters that need to be considered to have product impurities removed from a complex feed stream. Typically, parameters such as resin, ligand types, marker molecules, pH, and gradient profiles, as well as the sequence operation, have an enormous influence on the separation quality. The review article is very useful because it details the requirements and challenges associated with the data-driven, mechanistic, and hybrid modeling of chromatography.

The editors thank all those who have contributed to this Research Topic.
